# Peritoneal Fluid Transport rather than Peritoneal Solute Transport Associates with Dialysis Vintage and Age of Peritoneal Dialysis Patients

**DOI:** 10.1155/2016/8204294

**Published:** 2016-02-16

**Authors:** Jacek Waniewski, Stefan Antosiewicz, Daniel Baczynski, Jan Poleszczuk, Mauro Pietribiasi, Bengt Lindholm, Zofia Wankowicz

**Affiliations:** ^1^Nalecz Institute of Biocybernetics and Biomedical Engineering, Polish Academy of Sciences, 02 109 Warsaw, Poland; ^2^Military Institute of Medicine, 04 141 Warsaw, Poland; ^3^Divisions of Baxter Novum and Renal Medicine, Department of Clinical Science, Intervention and Technology, Karolinska Institutet, 141 52 Stockholm, Sweden

## Abstract

During peritoneal dialysis (PD), the peritoneal membrane undergoes ageing processes that affect its function. Here we analyzed associations of patient age and dialysis vintage with parameters of peritoneal transport of fluid and solutes, directly measured and estimated based on the pore model, for individual patients. Thirty-three patients (15 females; age 60 (21–87) years; median time on PD 19 (3–100) months) underwent sequential peritoneal equilibration test. Dialysis vintage and patient age did not correlate. Estimation of parameters of the two-pore model of peritoneal transport was performed. The estimated fluid transport parameters, including hydraulic permeability (LpS), fraction of ultrasmall pores (*α*_u_), osmotic conductance for glucose (OCG), and peritoneal absorption, were generally independent of solute transport parameters (diffusive mass transport parameters). Fluid transport parameters correlated whereas transport parameters for small solutes and proteins did not correlate with dialysis vintage and patient age. Although LpS and OCG were lower for older patients and those with long dialysis vintage, α_u_ was higher. Thus, fluid transport parameters—rather than solute transport parameters—are linked to dialysis vintage and patient age and should therefore be included when monitoring processes linked to ageing of the peritoneal membrane.

## 1. Introduction

The assessment of peritoneal transport in patients on peritoneal dialysis (PD) is usually based on directly measured indices such as net ultrafiltration, nUF, solute dialysate to plasma concentration ratio, D/P, free water transport, FWT, and clearances of small solutes and protein [1–6]. These indices typically comprise several transport processes; nUF is the combined effect of ultrafiltration to, and fluid absorption from, the peritoneal cavity, and D/P is the result of the combined effect of diffusive and convective solute transport. The separate transport components may be assessed by mathematical models. The model parameters characterize the function and indirectly also the structure of the peritoneal transport barrier by correlating flow rates to forces driving the transport. Therefore, mathematical models may provide insight into mechanisms of transport process. However, estimating the transport parameters for individual patients requires advanced clinical studies that go beyond the routine evaluation of peritoneal transport [2, 3, 5, 7].

The degree of generality and sophistication of transport models span from simple membrane models [8], through the three-pore (3p) or two-pore (2p) models [9–11], to the spatially distributed models [12–17]. The pore models of peritoneal fluid and solute transport may explain some seemingly puzzling phenomena, such as the discrepancy between the substantial sieving of small solutes and the low efficiency of low molecular osmotic agents (as glucose) expressed by the reflection coefficient, and were successfully applied for mathematical modeling of peritoneal transport [9, 18–26]. Except for the Personal Dialysis Capacity method [20, 27, 28], these models have in general not been applied for the estimation of parameters for individual patients, but, with the advent of the sequential peritoneal equilibration test (sPET), based on a simple clinical protocol, it is possible to estimate pore model parameters in individual patients [11, 26].

Using sPET and applying the 2p model, one may focus on fluid and small solute transport by assuming only two types of pores, ultrasmall and small pores [11], thus leaving out the third type of pores, large pores, through which according to 3p model macromolecules leak from blood to the peritoneal cavity [9, 29]. In this sPET plus 2p modeling approach, protein clearances are considered to be observational indices as they are not assessed by the 2p model [11]. Furthermore, the 3p model may not correctly describe the absorption of macromolecular volume markers from the peritoneal cavity and the effect of convective transport on albumin clearance [11, 22].

It is well established that peritoneal transport as assessed by observational indices such as nUF and D/P creatinine is changing with time on dialysis and ageing of the peritoneal membrane [30–36]. Recently, detailed mechanistic analyses of peritoneal changes during long-term PD were presented shedding light on underlying alterations in the pore system [35] although some indirect information was discussed previously [31]. In contrast, data on changes related to patients' age are scarce [37–39].

The aim of the current study was to explore to what extent changes of directly measured observational indices may reflect modifications of the pore system occurring with ageing of the peritoneal membrane related to advancing age of the patients and time on PD. For this purpose we estimated parameters of the 2p model, using data from sPET [11, 26], and investigated associations (1) between model parameters versus patient age and dialysis vintage, (2) between observational indices and estimated model parameters, and (3) between the model parameters themselves.

## 2. Methods

The study was carried out on 33 stable prevalent PD patients (15 females, 18 males) with mean age of 58.0 ± 16.8 (median 60, range 21–87) years and body weight of 77.4 ± 18.9 kg. The mean time on PD, “dialysis vintage,” was 26.1 ± 25.0 (median 19, range 3–100) months. None of the patients had peritonitis during or one month before the test. The test was performed together with the routine evaluation of membrane status. Written informed consent was obtained from each patient and the study was approved by the Ethical Committee of the Military Institute of Medicine, Warsaw, Poland.

The sPET consisted of two exchanges with different concentrations of glucose and dwell times, including standard peritoneal equilibration test (PET, glucose 2.27%, and 4 h) followed immediately by the mini peritoneal equilibration test (miniPET, glucose 3.86%, and 1 h [40]); see [26]. Each bag was weighed separately with and without infused and drained fluid and sampled for measurement of solute concentrations at the beginning and at the end of dialysis session. Dialysis fluid samples were taken also at 30 min of miniPET and 120 min of PET. One blood sample was collected at 120 min of PET. Sodium, glucose, urea, creatinine, phosphate, and albumin concentrations in plasma, fresh PD solution, and dialysate were analyzed using Cobas Integra 800 (Roche Diagnostics, Mannheim, Germany). The ionized sodium concentrations were measured using indirect ion selective electrode. IgM was measured by a nephelometric method.

The here applied 2p model is based on the same principles as the three-pore model; however, it describes only osmotically driven ultrafiltration, peritoneal absorption of fluid, and small solute transport while protein clearances are considered as independent parameters; protein transport is thus not included into the 2p model [11]. The two types of pores were considered, small pores of radius 43 Å and ultrasmall pores of radius 2 Å [19]; see [11] for the detailed description of the two-pore model.

Computer simulations were performed with the same parameter values for the data from PET and miniPET concurrently, and the parameters of the 2p pore model were estimated by adjusting the model predictions to clinical data. The diffusive mass transport coefficients were estimated separately for each small solute [26]. The effect of vasodilation was taken into account, as described in [41]. Osmotic conductance for glucose (OCG) was calculated as *σ*_G_ LpS, where *σ*_G_ was the reflection coefficient for glucose [19].

The Spearman correlation coefficient rho was used for the analysis of correlations. The two-variable linear regression was applied for the analysis of the relationship of the parameters versus patient age and dialysis vintage concomitantly. The statistical significance level was set at *p* = 0.05. Data are presented as mean ± standard deviation.

## 3. Results

**Figure 1:**
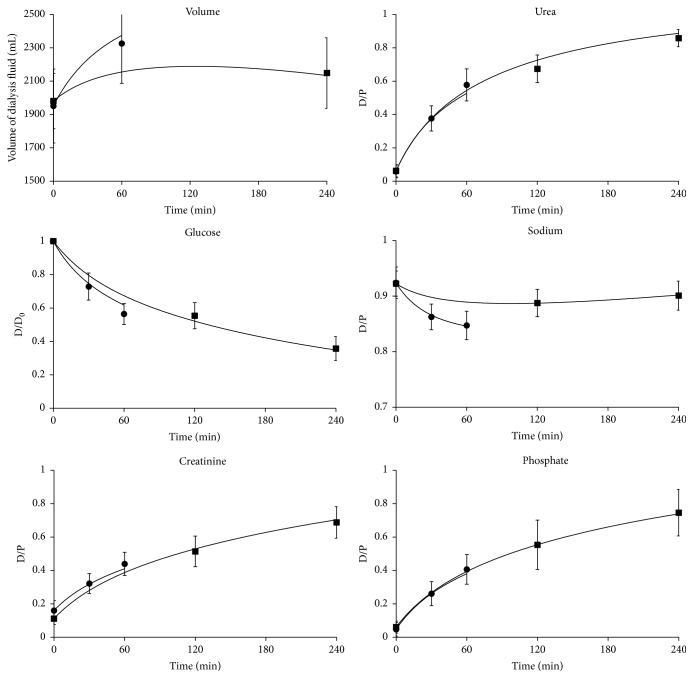
Measured values (mean ± SD) from PET (squares) and miniPET (circles) and best fit profiles provided by the two-pore (2p) model for dialysis fluid volume, dialysate to plasma concentration ratio (D/P) for urea, sodium, creatinine, and phosphate, and dialysate concentration over the initial concentration ratio (D/D_0_) for glucose.

**Figure 2:**
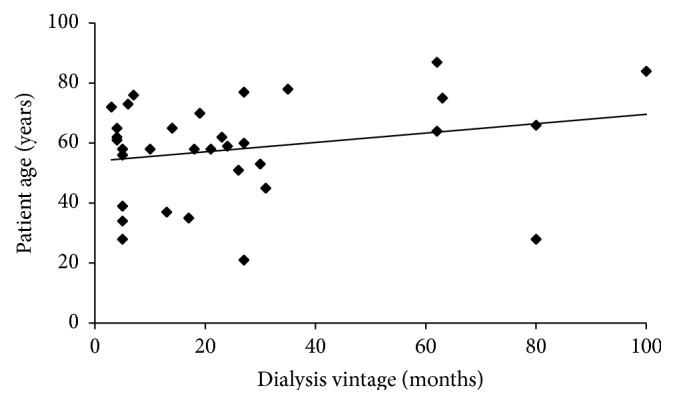
The relationship between dialysis vintage and patient age among 33 investigated patients undergoing peritoneal dialysis.

Data from PET and miniPET, representing a selection of parameters typically reported from these tests, are presented in Figure 1 and Table 1 together with their correlations to creatinine D/P on PET. Ultrafiltration volume and the ratio of dialysate glucose to initial dialysate glucose concentration (D/D_0_ glucose) on PET correlated negatively with PET D/P creatinine, as expected. A positive correlation with D/P for creatinine was found for D/P sodium in miniPET and protein clearances from both tests. The fluid transport parameters (nUF, small pore transport, and free water transport) correlated negatively with patient age, but no such correlation was found for the indicators of transport of small solutes and proteins, except for sodium D/P in miniPET that was found to depend on free water transport. The dialysis vintage did not correlate with patient age, rho = 0.197, and *p* = 0.27; see Figure 2. The dialysis vintage was in general not related to observational indices, except for the estimated ultrafiltration through small pores and free water fraction (Table 1). The measured mean dialysate volumes and solute D/P ratios in PET and miniPET are shown in Figure 1 together with the curves of the best fit of the 2p model.

### 3.1. Relationship of Transport Parameters to Patient Age and Dialysis Vintage

The hydraulic permeability, LpS, was found to decrease with age, while the fractional contribution of ultrasmall pores, *α*_u_, increased with age (Table 2). As a consequence, the reflection coefficient for glucose was higher for older patients; nevertheless, the osmotic conductance for glucose, OCG, was lower in older patients (Table 2). Among solute parameters, only the permeability-surface area coefficient (PS) for glucose was positively correlated with patient age and PS for urea with dialysis vintage. The fluid transport parameters correlated—in a similar direction—with dialysis vintage and patients' age: with increasing age and peritoneal dialysis vintage, respectively, LpS and OCG were lower and PA was lower (correlation only with vintage), whereas the fractional contribution of ultrasmall pores, *α*_u_, was higher (Table 2).

The application of the two-variable linear regression revealed only one observational parameter (among those listed in Table 1), namely, sodium D/P dip, that was significantly and concomitantly correlated with age (negatively) and dialysis vintage (positively) with adjusted *R* ^2^ = 0.23 (*p* = 0.008). Consequently, among the model parameters listed in Table 2, only *α*_u_ (positively), *α*_s_ (negatively), and *σ*_G_ (positively) correlated with both age and dialysis vintage (all with adjusted *R* ^2^ = 0.35; *p* = 0.001).

### 3.2. Correlations among Transport Parameters

The dip of dialysate sodium concentration, dip Na, correlated strongly positively with free water transport, FWT, and free water fraction, FWF, but negatively with ultrafiltration through small pores, UFSP, as expected, whereas no correlation was found with D/P creatinine (Table 3). In contrast, sodium D/P (at 1 h in miniPET) was positively correlated with D/P creatinine and negatively correlated with free water transport, FWT (Table 3). The clearances of proteins correlated with each other and with D/P creatinine (Tables 1 and 3).

The correlations between the model parameters show some mathematical coupling. As *α*_u_ + *α*_s_ = 1, the correlation with *α*_u_ means automatically reverse correlation with *α*_s_; compare Table 4. The correlation coefficients with *σ*_G_ are very close to those with *α*_u_; compare Table 4, because the values of *σ*_G_ are dominated by *α*_u_ [9]. LpS was correlated with PA (Table 4), probably because both of these fluid transport parameters depend on the surface area available for fluid transport. However, the fluid transport parameters LpS and PA were independent of diffusive small solute parameters, PS. The OCG correlated positively with LpS and *α*_s_ (Table 4).

Net UF in PET correlated negatively with PA and PS_G_ but did not correlate with LpS and OCG (Table 5). In contrast, nUF in miniPET correlated with LpS and OCG, but not with PS for small solutes (Table 5). FWF correlated strongly with *α*_u_ and negatively with OCG and LpS (Table 5). Dip Na correlated positively with *α*_u_ and negatively with PA (Table 5). Clearances of proteins correlated with PS for small solutes, but not with the parameters for fluid transport (Table 5).

## 4. Discussion

The main finding of the current study is that peritoneal fluid transport parameters—rather than solute transport parameters—are linked to factors potentially reflecting ageing of the peritoneal membrane, patient age, and dialysis vintage time. This underlines the importance of including peritoneal fluid transport parameters when monitoring peritoneal transport changes during long-term PD treatment.

In the current study, the 2p model described well the clinical data obtained by the sPET. The values of albumin and IgM clearances were similar to those found in other studies [42–46]. Model parameters that depend on the membrane surface area, as PS, LpS, and OCG, were found to be dependent on dwell time with initial values being about twice higher than the steady-state parameters shown in Table 2 (cf. [41]), whereas steady-state values are reached typically after about 2 hours [41] possibly reflecting at least in part the vasodilatory effect of dialysis fluids [17, 47]. Typically, transport parameters are assumed to be constant and may be thought of as representing a kind of average value; however, a good fit to the data was possible only when using time dependent parameters [22, 26].

In the current study, fluid transport parameters correlated with the age of the patients and dialysis vintage (though these two factors were not correlated), but in general no such dependency was observed for the transport of small solutes and proteins (Tables 1 and 2). In our cross-sectional study we did not find any consistent increase in small solute transport with dialysis vintage (except for urea PS, Table 2) that was observed in a few previous cohort studies [30–37]. The relations of fluid transport with patient age and dialysis vintage were similar suggesting that the time on PD had a similar effect on the peritoneal tissue transport system as patients' age. In general, fluid transport appeared to get worse with time, but lower hydraulic permeability is partly compensated by the higher fraction of ultrasmall pores (and therefore also the reflection coefficient for glucose, *σ*_G_; note that OCG = *σ*_G_ LpS and *σ*_G_ = *α*_u_ + 0.032*α*_s_); nevertheless, osmotic conductance, OCG, correlated negatively with both patients age and dialysis vintage (Table 2) suggesting that OCG may reflect the ageing of the peritoneal membrane. It is interesting to note that peritoneal absorption of fluid (PA) correlated negatively with dialysis vintage but not with patient age (Table 2) perhaps reflecting different effects of chronological and biological ageing on the systems involved in PA. The statistical independence of patient age and dialysis vintage (Figure 2) may be due to younger patients being on the treatment for a longer time; however, patients treated for long time were relatively few compared to those treated for short and medium time. An interesting interaction between age and dialysis vintage was found for sodium D/P dip, which did not correlate separately with each of them (Table 1), but in the two-variable regression correlated negatively with age but positively with dialysis vintage, which means that high sodium D/P dip was observed especially for young patient with long PD treatment.

The observed associations of patient age and dialysis vintage with the observational indices reflecting* functional* changes, and their interpretation by the mathematical model, do not necessarily correspond to characteristics and rate of* structural* changes. PD induces substantial modifications in the structure of the interstitium (fibrosis and thickening of the peritoneum), vasculopathy, loss of protective hyaluronan layer on the peritoneal surface of mesothelium, loss of mesothelial cells, and epithelial-to-mesenchymal transition [36, 48–50] that may result in loss of ultrafiltration capacity [1, 36]. The changes observed in the peritoneum of uremic patients who were investigated before dialysis initiation as well as in those on hemodialysis are much milder and appear at lower rate than changes in patients undergoing long-term treatment with PD [48].

With time on PD, small solute transport rates (assessed by PET) increase, nUF (due to fast dissipation of glucose osmotic gradient) decreases [30–36], and, according to some studies, hydraulic permeability may also decrease [31]. However, changes in small solute transport and nUF over time are reported to be dissociated; while there is a steady rate of increase in creatinine D/P, nUF is often stable during the initial years on PD and then suddenly drops [31]. Furthermore, changes in patients who finally developed loss of ultrafiltration capacity differ from those without this complication [31]. Other studies suggested that osmotic conductance remained relatively constant in patients with UF failure until a sudden drop occurred and the complication developed [51]. However, individual patients with UF failure may have much different patterns of changes in the pore transport characteristics before and after the onset of the complication [52].

The finding that PET and miniPET protein clearances correlated with PET D/P creatinine (Table 1) is in agreement with a previous study [37]; however, while 24 h albumin clearance in that study was stable with time on dialysis, age was among its predictors, but only during the initial period of follow-up [37].

In a unique study with 5-year follow-up of a cohort of incident patients undergoing standard peritoneal permeability analysis, SPA, there was a gradual decrease in nUF, FWT, and SPWT with time on dialysis [35]. Additionally, the basic parameters of the 3p model were estimated: radiuses of pores (with fixed *α*) and LpS, and the derived parameters were *σ* for glucose and OCG. No change in these derived parameters with time on dialysis was observed, in contrast to PS for creatinine that increased with dialysis vintage [35]. The methodology of that study did not allow, however, for the assessment of changes in the pore (*α*) parameters, and the contribution of ultrasmall pores, *α*_u_, was fixed for all patients and all tests in the same patient (*α*_u_ = 0.015). As data about values of pore radiuses and their change with time on dialysis were not presented, comparisons to our results are not possible at present.

Some of the observed relations between transport parameters and dialysis vintage in the current study differ from relations reported in other studies [30–36]; for example, dialysis vintage did not associate with PET creatinine D/P or PET nUF (Table 1). However, ultrafiltration through small pores and the fraction of fluid flow through ultrasmall pore (free water fraction) at miniPET correlated with dialysis vintage (Table 1). Furthermore, fluid transport parameters correlated with dialysis vintage: there was a mutually compensating decrease in LpS and PA and an increase in ultrasmall pore fraction *α*_u_ (which yields the increased reflection coefficient for glucose) (Table 2). The lower osmotic conductance of glucose with high dialysis vintage appeared to be compensated for by lower absorption of fluid from the peritoneal cavity, and this may explain why nUF did not associate with dialysis vintage (Tables 1 and 2).

Some parameters of fluid transport, as peritoneal absorption, PA, and osmotic conductance of glucose, OCG, can also be measured independently of the estimation of pore model parameters [8, 53, 54]. In agreement with our results that PA and OCG are independent of direct indicators of small solute transport (as D/P creatinine, Table 5) and of small solute PS (Table 4), these direct assessments also demonstrated that PA and OCG are independent of D/P creatinine [55]. However, as shown in the present study, basic parameters of fluid transport (LpS, *α*, and *σ*_G_) correlated with D/P creatinine (Table 5). Therefore, restricting analyses only to OCG could potentially hide features of the pore system that may associate with changes in the membrane structure.

The peritoneal absorption, PA, describes the rate of fluid absorption from the peritoneal cavity [1–6]. It comprises the absorption to lymphatic vessels open to the peritoneal cavity (mostly in the diaphragm) and the absorption to the tissue of abdominal organs and muscles that is driven by the increased hydrostatic pressure in the peritoneal cavity. The absorption is in particular responsible for the decline of dialysis fluid volume after its osmotic force is dissipated [1–6]. In the 2p model, PA is a constant independent parameter and its values estimated in our study are in agreement with those found in other clinical studies with different volume markers or by mathematical modeling [5, 14].

Limitations of the current study include the observational cross-sectional design which precludes ascertainment regarding longitudinal changes and conclusions regarding causality; the relatively small number of patients; the lack of detailed analysis of leakage of macromolecules from blood to the peritoneal cavity; and lack of data on biomarkers of peritoneal health. On the other hand, all investigated patients underwent a uniform protocol with concurrent measurements of transport indices and estimations of fluid and small solute transport parameters derived from sPET, that is, the combination of conventional PET followed by miniPET.

We conclude that the new approach of combining sPET for the collection of clinical data on peritoneal transport with estimation of transport parameters based on the modified 2p model of peritoneal transport is a feasible method that provides values of transport parameters in line with previous studies and may provide mechanistic insights into sPET data. The main findings are that fluid transport parameters (PA, LpS, and OCG) were independent of solute transport parameters (PS) and that whereas fluid transport parameters correlated with patient age and dialysis vintage, such associations were not observed for transport parameters for small solutes and proteins. The resemblance in the changes of transport patterns with increasing age and long time on PD suggests an analogous effect of dialysis vintage and uremic patient's age on the peritoneal transport barrier and links to the increasing awareness of premature ageing in chronic kidney disease [56].

## Figures and Tables

**Table 1:** Peritoneal transport characteristics (mean ± SD) from miniPET (after 1 h dwell) and PET (after 4 h dwell) and their correlations (expressed as Spearman correlation coefficient rho) with creatinine D/P, patient age, and dialysis vintage, for 33 prevalent peritoneal dialysis patients.

Parameters	Parameter value	Correlation with creatinine D/P	Correlation with age	Correlation with dialysis vintage
*miniPET*				
Net ultrafiltration [mL]^a^	375 ± 107	NS	−0.62	NS
Ultrafiltration through small pores^b^ [mL]	168 ± 107	NS	−0.36	−0.37
Free water transport [mL]^c^	207 ± 62	NS	−0.41	NS
Free water fraction [%]^d^	58 ± 20	NS	NS	0.37
D/P sodium	0.85 ± 0.03	0.57	0.38	NS
Dip D/P sodium	0.077 ± 0.028	NS	NS	NS
Albumin clearance	0.127 ± 0.049	0.68	NS	NS
*PET*				
Net ultrafiltration [mL]	168 ± 168	−0.47	−0.53	NS
D/P creatinine	0.66 ± 0.10	1.00	NS	NS
D/D_0_ glucose	0.36 ± 0.07	−0.85	NS	NS
Albumin clearance	0.099 ± 0.038	0.78	NS	NS
IgM clearance	0.020 ± 0.013	0.58	NS	NS

^a^Net ultrafiltration (netUF60) was defined as the difference between the weight of the effluent (Vend) and the weight of the infused peritoneal dialysis fluid (V0); ^b^ultrafiltration through small pores UFSP60 = RemNa/CBNa, where sodium removal RemNa = Vend *∗* CNaEnd − V0 *∗* CNa0; ^c^free water transport FWT = netUF60 − UFSP60; ^d^free water fraction FWF = FWT/netUF60.

D/P sodium, dialysate to plasma sodium concentration (D/PNa) at the end (D/PNa60) of miniPET; dip D/P sodium, sodium dip DipNa60 = D/PNa0 − D/PNa60; NS, not significant.

**Table 2:** Parameters of the pore model (mean ± SD) estimated from sPET data using the 2p model and the correlation coefficients rho for the correlation of the parameters of the 2p model with patient age and dialysis vintage in 33 prevalent patients undergoing peritoneal dialysis.

Parameters	2p model	Correlation with age	Correlation with dialysis vintage
*Fluid transport*			
LpS [mL/min/mmHg]	0.033 ± 0.022	−0.46	−0.59
PA [mL/min]	1.3 ± 0.95	NS	−0.55
*α*_u_	0.07 ± 0.07	0.35	0.55
*α*_s_	0.93 ± 0.07	−0.35	−0.55
*σ*_G_	0.104 ± 0.066	0.35	0.55
OCG [mL/min/mmHg]	0.0023 ± 0.0008	−0.47	−0.52
*Solute transport*			
PS_G_ [mL/min]	7.7 ± 2.3	0.36	NS
PS_Na_ [mL/min]	4.2 ± 3.5	NS	NS
PS_U_ [mL/min]	15.9 ± 3.7	NS	0.37
PS_Cr_ [mL/min]	8.0 ± 2.8	NS	NS
PS_P_ [mL/min]	9.5 ± 3.3	NS	NS

LpS, hydraulic permeability; PA, peritoneal absorption rate; *α*_usmall_ and *α*_small_, fractional contribution of ultrasmall and small pores, respectively, to LpS; PS_G_, PS_Na_, PS_U_, PS_Cr_, PS_P_, and PS_A_, diffusive mass transport coefficients for glucose (G), sodium (Na), urea (U), creatinine (Cr), and phosphate (P) (these parameters were estimated from clinical data); *σ*_G_, reflection coefficient for glucose, and OCG, osmotic conductance for glucose, were calculated as described in Methods; NS, not significant.

**Table 3:** Correlation coefficients rho for observational parameters from miniPET and PET. The values of rho are shown if *p* < 0.05.

	UF miniPET	UF PET	UFSP	FWT	FWF	D/P Cr	D/D_0_ G	D/P Na	Dip Na	Cl Alb miniPET	Cl Alb PET	Cl IgM PET
UF miniPET	1.00	·	·	·	·	·	·	·	·	·	·	·
UF PET	NS	1.00	·	·	·	·	·	·	·	·	·	·
UFSP	**0.82**	NS	1.00	·	·	·	·	·	·	·	·	·
FWT	NS	**0.49**	NS	1.00	·	·	·	·	·	·	·	·
FWF	**−0.53**	NS	**−0.90**	**0.65**	1.00	·	·	·	·	·	·	·
D/P Cr	NS	**−0.47**	NS	NS	NS	1.00	·	·	·	·	·	·
D/D_0_ G	NS	NS	NS	NS	NS	**−0.85**	1.00	·	·	·	·	·
D/P Na	NS	**−0.43**	NS	**−0.52**	NS	**0.57**	**−0.45**	1.00	·	·	·	·
Dip Na	NS	**0.40**	**−0.51**	**0.89**	**0.74**	NS	NS	**−0.43**	1.00	·	·	·
Cl Alb miniPET	NS	NS	NS	**−0.38**	NS	**0.68**	**−0.57**	**0.55**	NS	1.00	·	·
Cl Alb PET	NS	NS	NS	NS	NS	**0.78**	**−0.69**	**0.50**	NS	**0.85**	1.00	·
Cl IgM PET	NS	NS	NS	NS	NS	**0.58**	**−0.42**	NS	NS	**0.64**	**0.76**	1.00

For abbreviations, see Tables 1 and 2.

**Table 4:** Correlation coefficients rho for parameters of the modified 2p model. Values of rho are shown if *p* < 0.05.

	LpS	PA	*α*_u_	*α*_s_	*σ*_G_	OCG	PS_U_	PS_G_	PS_Na_	PS_Cr_	PS_P_
LpS	1.00	·	·	·	·	·	·	·	·	·	·
PA	**0.72**	1.00	·	·	·	·	·	·	·	·	·
*α*_u_	**−0.88**	**−0.64**	1.00	·	·	·	·	·	·	·	·
*α*_s_	**0.88**	**0.64**	−1.00	1.00	·	·	·	·	·	·	·
*σ*_G_	**−0.88**	**−0.64**	1.00	−1.00	1.00	·	·	·	·	·	·
OCG	**0.87**	**0.64**	**−0.61**	**0.61**	**−0.61**	1.00	·	·	·	·	·
PS_U_	NS	NS	NS	NS	NS	NS	1.00	·	·	·	·
PS_G_	NS	NS	NS	NS	NS	NS	**0.71**	1.00	·	·	·
PS_Na_	NS	NS	**0.46**	**−0.46**	**0.46**	NS	**0.42**	**0.61**	1.00	·	·
PS_Cr_	NS	NS	**0.38**	**−0.38**	**0.38**	NS	**0.74**	**0.90**	**0.67**	1.00	·
PS_P_	NS	NS	NS	NS	NS	NS	**0.69**	**0.88**	**0.68**	**0.84**	1.00

For abbreviations, see Tables 1 and 2.

**Table 5:** Correlation coefficients rho for observational parameters from miniPET and PET versus parameters of the robust 2p model. Values of rho are shown if *p* < 0.05.

	LpS	PA	α_u_	α_s_	σ_G_	OCG	PS_U_	PS_G_	PS_Na_	PS_Cr_	PS_P_

UF miniPET	**0.79**	**0.45**	**−0.54**	**0.54**	**−0.54**	**0.88**	NS	NS	NS	NS	NS
UF PET	NS	**−0.55**	NS	NS	NS	NS	NS	**−0.46**	NS	**−0.41**	**−0.42**
UFSP	**0.76**	**0.59**	**−0.67**	**0.67**	**−0.67**	**0.75**	NS	NS	NS	NS	NS
FWT	NS	NS	NS	NS	NS	NS	NS	**−0.35**	NS	NS	**−0.37**
FWF	**−0.63**	**−0.57**	**0.70**	**−0.70**	**0.70**	**−0.52**	NS	NS	NS	NS	NS
D/P Cr	**−0.34**	NS	**0.39**	**−0.39**	**0.39**	NS	**0.62**	**0.85**	**0.72**	**0.89**	**0.73**
D/D_0_ G	NS	NS	NS	NS	NS	NS	**−0.49**	**−0.70**	**−0.69**	**−0.70**	**−0.66**
D/P Na	NS	NS	NS	NS	NS	NS	NS	**0.66**	**0.51**	**0.58**	**0.64**
Dip Na	NS	**−0.41**	**0.43**	**−0.43**	**0.43**	NS	NS	NS	NS	NS	NS
Cl Alb miniPET	NS	NS	NS	NS	NS	NS	**0.53**	**0.69**	**0.49**	**0.64**	**0.64**
Cl Alb PET	NS	NS	NS	NS	NS	NS	**0.60**	**0.80**	**0.58**	**0.73**	**0.70**
Cl IgM PET	NS	NS	NS	NS	NS	NS	**0.49**	**0.57**	**0.37**	**0.61**	**0.51**

For abbreviations, see Tables 1 and 2.
